# Cognitive Aging in Zebrafish

**DOI:** 10.1371/journal.pone.0000014

**Published:** 2006-12-20

**Authors:** Lili Yu, Valter Tucci, Shuji Kishi, Irina V. Zhdanova

**Affiliations:** 1 Department of Anatomy and Neurobiology, Boston University School of Medicine Boston, Massachusetts, United States of America; 2 Department of Cancer Biology, Dana Farber Cancer Institute, Harvard Medical School Boston, Massachusetts, United States of America; University of Birmingham, United Kingdom

## Abstract

**Background:**

Age-related impairments in cognitive functions represent a growing clinical and social issue. Genetic and behavioral characterization of animal models can provide critical information on the intrinsic and environmental factors that determine the deterioration or preservation of cognitive abilities throughout life.

**Methodology/Principal Findings:**

Behavior of wild-type, mutant and gamma-irradiated zebrafish (*Danio rerio*) was documented using image-analysis technique. Conditioned responses to spatial, visual and temporal cues were investigated in young, middle-aged and old animals. The results demonstrate that zebrafish aging is associated with changes in cognitive responses to emotionally positive and negative experiences, reduced generalization of adaptive associations, increased stereotypic and reduced exploratory behavior and altered temporal entrainment. Genetic upregulation of cholinergic transmission attenuates cognitive decline in middle-aged *achesb55/+* mutants, compared to wild-type siblings. In contrast, the genotoxic stress of gamma-irradiation accelerates the onset of cognitive impairment in young zebrafish.

**Conclusions/Significance:**

These findings would allow the use of powerful molecular biological resources accumulated in the zebrafish field to address the mechanisms of cognitive senescence, and promote the search for therapeutic strategies which may attenuate age-related cognitive decline.

## Introduction

Cognitive abilities are rapidly established during development, stabilize in adults and may decline with aging. This general trend has its notable exceptions, represented by the early onset of cognitive deficits in some individuals, in contrast to others who preserve remarkably high cognitive functions until very old age. A combination of genetic, environmental and social factors is believed to contribute to this diversity. Furthermore, cognitive status in early life is suggested to be an important predictor of late-life cognitive abilities [1]. Thus, understanding the specific roles and interactions between genetic and environmental factors or individual experiences requires analysis of cognitive changes throughout the entire life. The importance of studying life-long changes in cognitive functions, though acknowledged, has not yet been adequately addressed [2].

Animal models have long been recognized as a necessary means to study complex biological processes, especially those on the level of integrative functions. Behavioral, morphological or neurophysiological correlates of aging are extensively studied in diverse organisms, ranging from caenortabitidis (*C. elegans*) to non-human primates [3], [4]. Each animal model of human aging has its inherent advantages and limitations, since no single model exactly reflects the age-related processes that occur in humans. Most likely, only a combination of data collected in different models will help to decipher the principal factors that contribute to age-dependent cognitive decline in our species. Use of vertebrate models subject to detailed and high-throughput molecular biological analysis, and large-scale mutagenesis approaches, might be pivotal in both understanding the mechanisms of age-related cognitive decline, and in searching for therapeutic strategies to attenuate it.

Recently, the zebrafish (*Danio rerio*) has attracted attention as a potential model of aging [5]–[7]. The unique advantages of zebrafish, long-appreciated by developmental biologists and geneticists, stem from the specific features of this vertebrate. Every week a female zebrafish can spawn hundreds of transparent eggs which when externally fertilized contain transparent embryos. This provides numerous undisturbed windows into the development of all the vertebrate structures and systems. The rapid development of zebrafish, which hatch from fertilized eggs around 50 hours post-fertilization, and actively swim and hunt for prey three days later, allows early-life screens for behavioral trends in hundreds of 4 mm long vertebrate siblings. Vast information on zebrafish development, molecular biology and genetics, successful large-scale mutagenesis programs and the availability of multiple mutant and transgenic phenotypes, make zebrafish uniquely suitable for studying genetic and early developmental factors that affect the aging process in vertebrates.

Furthermore, uninterrupted growth, without undergoing metamorphosis, provides a continuum of development, maturation and aging in zebrafish. This may allow the studying of age-related processes as they gradually unfold. Considering the high restorative capacity in fishes, compared to mammals, monitoring such processes could bring insights into powerful compensatory mechanisms promoting “successful aging” in vertebrates, or those inducing early cognitive and physical impairments.

A relatively long life span in zebrafish (3–5 years) could be viewed as a technical disadvantage for aging research, compared to such short-lived genetic animal models as *C. elegans*, Drosophila or even mice. However, this factor may hold certain promise [5], providing a model of very gradual rather than rapid decline in specific physiological functions and systems, similar to that observed in humans. This may also assist in studying the role of mild environmental factors in the aging process, which might not be easily recognized in short-lived species due to their subtle and gradual cumulative effects. Such environmental factors, along with acute and strong toxicities, may play an important role in the rate of human senescence.

Studying the impact of environmental factors and pharmacological agents in zebrafish is relatively easy. These animals have well-developed sensory organs, detect diverse environmental stimuli, and show well-defined behavioral responses to them. Zebrafish skin or gills provide a gateway for many soluble agents. As a result, the majority of biologically active compounds can be administered non-invasively, simultaneously and in precise concentrations directly into the water surrounding hundreds of embryos, larvae or adult zebrafish.

Zebrafish have complex social interactions, being a shoaling fish [8]–[10]. Similar to humans, individual and social behavioral trends and the social environment may significantly affect zebrafish health, cognitive functions and rate of aging. The ability to study an impact of the social roles or strategies in the aging process, in a prolific vertebrate model is difficult to overestimate. High-throughput image analysis techniques now provide new opportunities for carrying out behavioral screens in zebrafish, by documenting spontaneous or induced behaviors in hundreds of larval fish or dozens of adults. This methodological approach, allowed the characterization of circadian rhythms [11] and sleep [12] in zebrafish, and can now also be applied to the study of zebrafish social interactions.

To characterize several aspects of cognitive aging in zebrafish, we used young, middle-aged and old fish (1, 2 and 3 years of age, respectively). A battery of tests assessing their ability to adapt to new temporal, spatial, and visual cues was employed. New timing of restricted food administration served as a temporal cue. The side of the tank where an unconditioned stimulus (US) was administered served as a spatial cue. The red color of the walls of the experimental tank or T-maze served as a visual cue.

**Figure 1:**
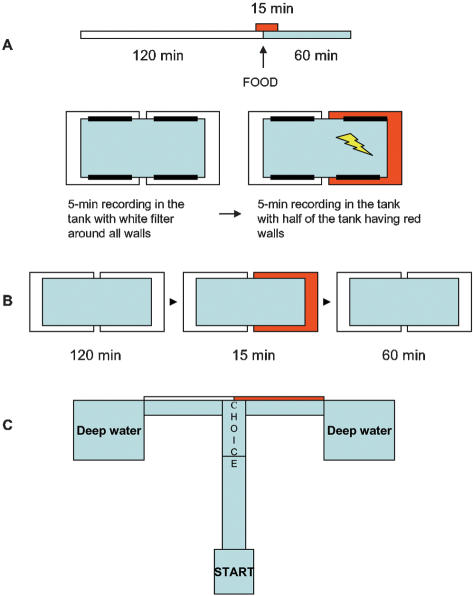
Schematic illustrations of the methodological approaches used. A. Time schedule of CPP procedure: 120 min of baseline recording without food in all-white tank, followed by 60 min of recording after food administration. Red color was present for 15 min, starting 5 min prior to food administration. B. CPA procedure: Each day, zebrafish behavior was recorded first in the test tank with all white walls (5 min), then for 5 min after half of the tank walls changed to red color. Horizontal black bars - stimulating electrodes. Electric shock (yellow “lightening”) delivered only in the red zone. C. T-maze with Start and Choice zones, inserted color filters and deep water end compartments. The apparatus (30 mm water level) included a starting zone, 40 × 30 mm, separated from the rest of the maze by a transparent removable partition. Behind the partition, there was a long 80 × 20 mm arm and two short 60 × 20 mm arms, which lead to the removable deep water chambers (85 mm water level).

Considering the critical roles of emotion and motivation in the learning process and in memory retention, we used both positive and negative reinforcement while conditioning zebrafish to new experimental paradigms. In the conditioned place preference paradigm (CPP), the red color of one side of the tank was associated with food presentation (Fig. 1A). In the conditioned place avoidance (CPA) paradigm, the side of the tank with the red-colored walls was the one where the fish received a mild electric shock (Fig. 1B).

We have also explored whether zebrafish can generalize a developed response to a conditioned stimulus (CS) and apply its meaning to either prefer or avoid the red color in a novel environment, T-maze apparatus (Fig. 1C).

The activity of the cholinergic system is suggested to be an important factor in human cognitive aging [1], [13], [14]. To investigate whether this system may also play a role in zebrafish behavior, young and middle-aged *achesb*55/+ mutants (referred to later as ACHE mutants) with increased acetylcholine levels due to impairment in acetylcholinesterase function [15] were tested. Their cognitive performance was compared to the wild-type siblings.

Based on our earlier observations that gamma-irradiated fish display some molecular and physiological signs of accelerated aging [16], we explored whether this genotoxic stress would also result in cognitive alterations resembling those of aged animals. To address this question, young zebrafish were subject to gamma-irradiation (IR) seven months before their cognitive performance was assessed.

Here we present an initial characterization of the age-related cognitive changes of zebrafish, and conclude that this organism may become an important player in understanding cognitive aging. Our data indicate that aging in zebrafish is associated with changes in cognitive responses to spatial, visual and temporal cues, reduced generalization of adaptive associations. The data also suggest that aged zebrafish may have increased stereotypic and reduced exploratory behavior. The results obtained in *achesb*55/+ mutant zebrafish with increased acetylcholine levels, and in fish that have undergone genotoxic stress, demonstrate potential opportunities of using this species to study the mechanisms involved in the delay or acceleration of cognitive aging.

## Results

### Entrainment to a temporal cue

Temporal organization of physiological functions depends on both the intrinsic biological clock and the entrainment to environmental cues. While the light-dark cycle is the major environmental factor for circadian entrainment, the timing of restricted food availability can also powerfully synchronize animals' behavior [17], [18]. Furthermore, these distinct environmental cues appear to involve partially independent circadian oscillators. To examine whether zebrafish of different ages can demonstrate food entrainment, at the start of the Adaptation period their feeding schedule was changed to a new time and restricted to one hour per day.

**Figure 2:**
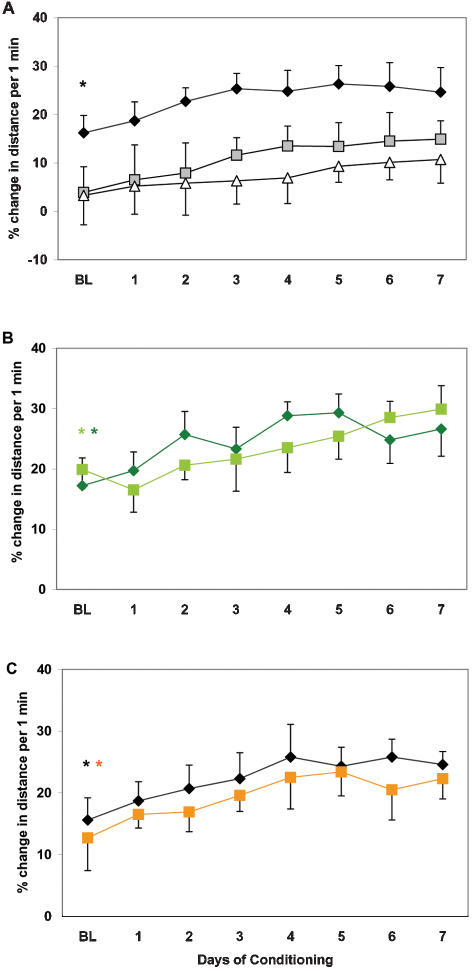
Development of anticipatory increase in locomotor activity prior to food administration in wildtype (A) and ACHE mutants (B) of different age, and in IR zebrafish (C). X-axis: % change in activity level within 30 min prior to food administration, as compared to preceding 90-min period. First day of significant (p<.05) change in group behavior in young (*) and middle-aged (**) fish. Note that BL was assessed after 7 days of new timing of daily food administration in white/white environment. Young fish – diamond, middle-aged fish – square, old fish – triangle. Wildtype – black; ACHE mutants – green; IR zebrafish – orange.

Young age correlated with the ability of wild type zebrafish to anticipate a meal. Following 7 days of new timing of feeding, (i.e., at Baseline), their locomotor activity was significantly higher 30 min prior to food administration, compared to the preceding 90-min period (p<0.01; Fig. 2A). This difference remained significant throughout the rest of the experiment (p<0.05). Several middle-aged and old fish also showed an anticipatory increase in activity. However, as a group, they failed to significantly entrain to the new time of feeding.

The wild type siblings of mutant fish showed an age-dependent decline in food anticipation, similar to the AB wildtype strain. However, middle-aged ACHE mutants did not differ from young mutants in this parameter (Fig. 2B). Both mutant age groups significantly increased their activity close to the time of food administration (p<0.05).

Young zebrafish subject to gamma-irradiation (IR fish) showed fast development of the anticipatory reaction to the new timing of food intake and, similar to the age-matched control, showed significant anticipation at Baseline (Fig. 2C).

These data suggest that zebrafish can efficiently entrain to a new time of food administration, showing an anticipatory increase in locomotor activity. Such food entrainment declines with aging in wild-type zebrafish, but is attenuated in the animals with an upregulated cholinergic system. Gamma-irradiation does not significantly affect food entrainment 7 months after exposure.

### Entrainment to a spatial cue

During the Adaptation and Baseline periods, food was administered into the middle of the tank. At the end of the Baseline period, crossing of the middle zone of the tank within an hour prior to feeding increased in young wildtype fish and in mutants of both age groups. However, this parameter did not reach the level of significance in any of the group tested. No tendency toward spending more time in the middle of the tank was observed in middle-aged, old or IR fish.

During the Conditioning period, food was administered in one designated side of the tank, right or left, and associated with the red-colored wall. During the Extinction period, food was again administered to the middle of the tank.

**Figure 3:**
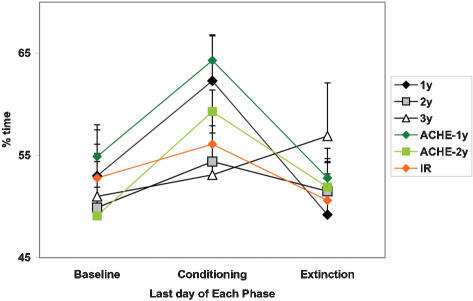
Spatial entrainment in zebrafish groups. Percent time spent in the US-associated side of the tank during food anticipation, i.e., 30 min interval prior to CS presentation, at the end of Baseline, Conditioning and Extinction phases. Young fish (1 y) – diamond, middle-aged (2 y) fish – square, old fish (3 y) – triangle. Wildtype – black; ACHE mutants – green; IR zebrafish – orange.

At Baseline, no significant difference in time spent in the left/right or red/white zones of the tank was documented between the experimental groups. After six days of Conditioning, the food anticipatory reaction (as described above) in young fish and ACHE mutants of both ages became significantly (p<0.05) associated with the side of the tank where food was administered, as assessed during the 30-minute period prior to red color presentation (Fig. 3).

In contrast, as a group, middle-aged and old fish failed to show a significant change in activity or side-preference prior to the administration of CS. However, several individual fish of the two older groups demonstrated both anticipation and preference of the food-associated side of the tank. Except one animal, these fish were the same older individuals that showed early onset of a conditioned response to red color.

Although the IR fish showed unaltered food entrainment, there was no significant association of this behavior with the side of the tank. Thus, IR fish failed to develop a response to the spatial cue.

Throughout the Extinction period, preference for one side of the tank during food anticipation gradually declined in young wild-type and mutant fish. No significant preference for the middle of the tank developed during this short period.

### Conditioned place preference (CPP) in positive reinforcement paradigm

#### CPP in wild-type zebrafish

**Figure 4:**
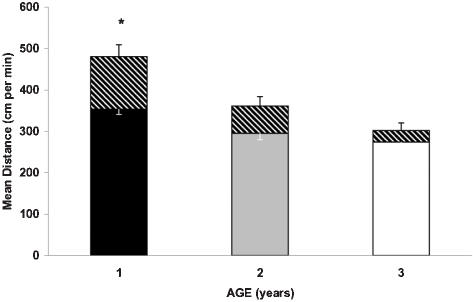
Baseline locomotor activity level is significantly reduced in middle-aged (2 years old) and old (3 year old) fish, compared to young (1 year old) zebrafish. Y axis: mean distance traversed. Solid color of each bar – slow motion; diagonal part –fast motion. * p<0.05 for comparisons between young and middle-aged, and p<0.001 between young and old zebrafish. No significant difference between two older groups.

At Baseline, young fish had significantly higher activity levels, compared to middle aged and old zebrafish (p<0.05 and p<0.01, respectively). This was reflected in the distance traversed, with both slow-motion and fast-motion distance being higher in young animals (Fig. 4). No significant baseline difference in the overall activity level or a ratio of slow/fast-motion was documented between middle-aged and old fish.

**Figure 5:**
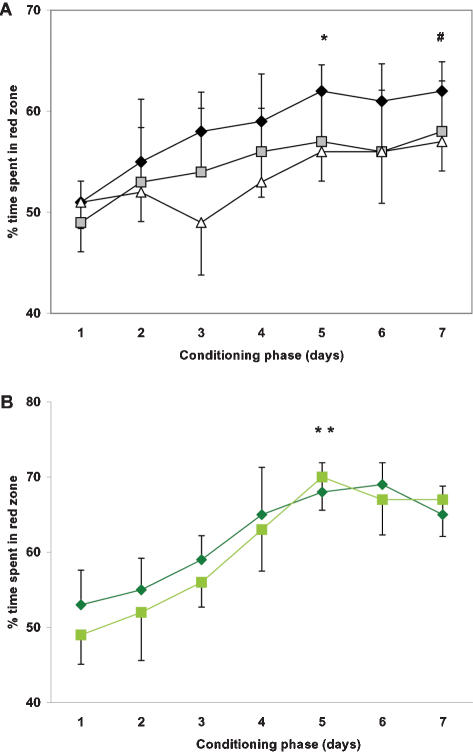
Learning curves in fish of different age: wildtype (A), ACHE mutants (B). Increase in time spent in the red zone during 5 min of red color presentation prior to food administration, compared to the same group behavior at Day 1. Young fish – diamond, middle-aged fish – square, old fish – triangle. First day of significant change in group behavior, * p<0.01 in young wildtype and mutant fish. # p<.05 in middle-aged wildtype.

During the Conditioning phase, the percentage of time spent in the context-conditioned environment after the CS was presented (i.e., red zone prior to food administration) was inversely correlated with age. The repeated measures ANOVA, followed by post-hoc analysis revealed a significant (Newman Keuls: p = 0.001) increase in the time spent in the context-conditioned part of the tank for the young group, compared to both middle-aged and old zebrafish (Fig. 5A). On Day 5 of conditioning, young fish spent significantly more time in the red zone of the tank, compared to Baseline (p<0.01). In contrast, in the middle-aged fish, the tendency to spend more time in the red zone reached statistical significance only after 7 days of conditioning (p<0.05). No significant change was documented in the behavior of aged fish, though there was a trend towards increased time in the red zone. It should be noted, however, that some of the middle-aged and even old fish were capable of learning the paradigm as fast as typical young fish, while the majority of old fish did not show color preference throughout the Test period.

#### CPP in achesb55/+ zebrafish

The groups of 1- and 2-year old ACHE mutants were compared to their wild-type siblings. All the fish groups tested significantly increased time spent in the red zone by the fifth day of Conditioning (p<0.05). No significant difference in Baseline locomotor activity was observed between the young wild-type siblings and ACHE mutants of both ages. Similarly, no significant difference was observed between young wild-type siblings and ACHE mutants of both ages during the Conditioning phase of the study. In contrast to 2-year old wild-type siblings, middle-aged ACHE mutants did not significantly differ from young ACHE mutants in any of the parameters assessed (Fig. 5B). No significant difference between the AB-strain and wild-type siblings of ACHE mutants of both ages was documented (data not shown).

#### CPP in young gamma irradiated fish

**Figure 6:**
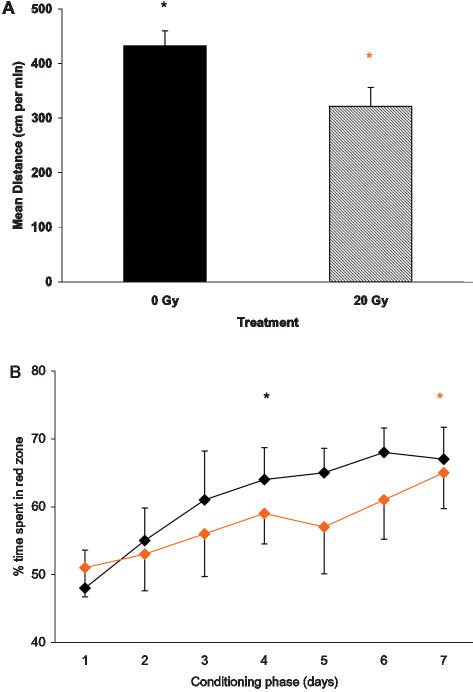
Changes in performance in young zebrafish 7 months after whole body gamma-irradiation, compared to same age control. A: Reduction in locomotor activity in the irradiated zebrafish (distance traversed). B: Reduction in developing CPP; time spent in the red half of the tank during 5 min of red color presentation prior to food administration. * 0 Gy or * 20 Gy p<0.05, in A: relative to Control; in B: relative to day 1 for the same group.

Comparison of 1-year old zebrafish that received 20 Gy irradiation 7 months prior to behavioral assessment to age-matched control fish (0 Gy), showed a statistically significant difference between the groups according to repeated measures ANOVA. The locomotor activity in the irradiated zebrafish was lower, including the distance travelled and speed (Newman Keuls: p<0.05; Fig. 6A). These fish also showed a delay in developing a conditioned color preference, similar to middle-aged fish (Fig. 6B).

#### Extinction and reinstatement of CPP

**Figure 7:**
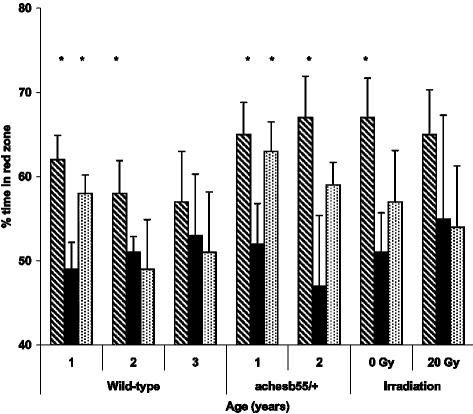
Change in place preference during and following Extinction. Percent time spent in the red half of the test tank 5 min prior to food administration on the last day of Conditioning (day 7, diagonal bar), same side of the tank on the last day of Extinction (day 11 in white-white test tank; black bar) and during post-extinction presentation of red color (day 12; dotted bar). Y-axis: % time spent in the red half of the tank (or corresponding half on day 11). X-axis – zebrafish age. * p<0.05 relative to each group's end of Extinction, day 11.

During the Extinction phase, food was administered into the middle of the tank, rather than on one side, and this was not associated with the red color presentation. The young wild-type zebrafish and mutants of both ages showed a decline in side preference during Extinction (p<0.05; Fig. 7). When red color was reintroduced following the Extinction period (day 12), young wild-type fish (AB-strain and siblings of the mutant fish), as well as ACHE mutants of both ages, showed increased preference for the red side of the tank, compared to the last day of Extinction (day 11), when this side of the tank was coloured white (p<0.05; Fig. 7). However, the side preference was less pronounced than at the end of the Conditioning phase and did not reach the level of significance in middle-aged ACHE mutants and wild-type siblings of irradiated fish. No increase in the time spent on the red side of the tank was observed in the two older groups of wild-type and young irradiated zebrafish following the Extinction period.

#### Generalization of CPP to different environment

To determine whether zebrafish can generalize a CS to a new environment, fish were tested in a T-maze following each phase of the study. The color of the two short arms of the maze, leading to deep water chambers, was either the same (white/white) or different (red/white). No food reinforcement was provided in the T-maze. The choice of left-right or white-red arm was documented over 14 consecutive trials.

**Figure 8:**
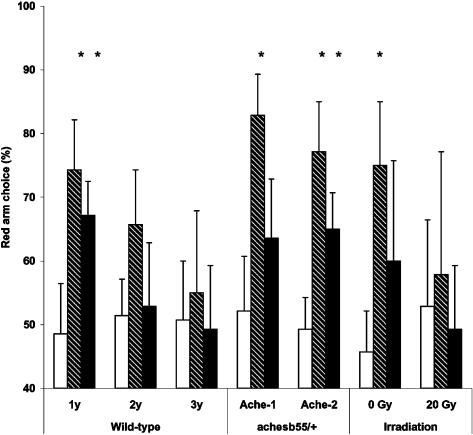
Generalization of red color preference in a “different condition” of the T-maze at Baseline, following Conditioning and Extinction phases of CPP paradigm. Y-axis: percent of red arm choices over 14 consecutive trials. X-axis – zebrafish age/strain. White bar – Baseline; diagonal pattern – after Conditioning, black – after Extinction. * p<0.05 relative to each group Baseline.

Young wild-type zebrafish and ACHE mutants of both ages demonstrated significant increase in the preference of a red-colored arm of the T-maze following the Conditioning phase, as compared to Baseline (p<0.05; Fig. 8). The middle-aged wild-type fish also increased their choice of the red arm of the maze after Conditioning, but this effect did not reach statistical significance. The group of old wild-type fish and irradiated young fish did not show color preference.

Following the Extinction phase, preference of red color in the T-maze remained significant in young wild-type (both AB strain and mutant siblings) and middle-aged ACHE mutants (p<0.05; Fig. 8). However, a group of young wild-type zebrafish serving as a control to irradiated fish and young ACHE mutants did not retain significant color preference in T-maze following Extinction phase.

#### Age differences in activity and arm choice in T-maze

**Figure 9:**
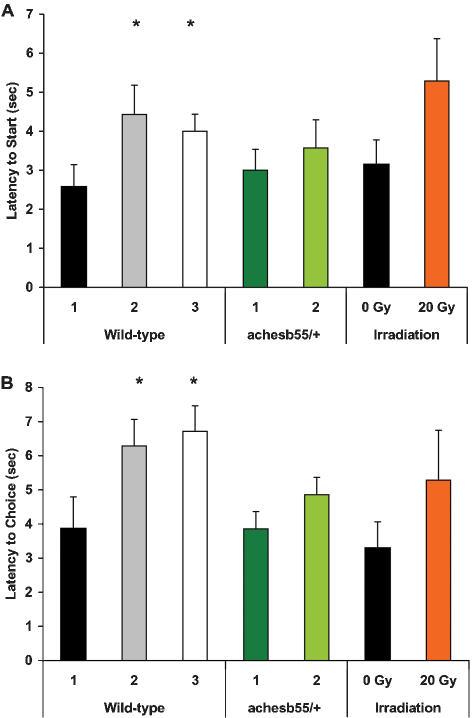
Latency to Start (A) and Choice (B) at Baseline T-maze test in different groups of zebrafish. X-axis – fish age (years) and treatment. * p<0.01, relative to young control of the same strain or treatment group.

Locomotor activity levels and the time spent in each area of the T-maze revealed certain differences between the age groups. Middle-aged and old wild-type zebrafish demonstrated a significantly longer latency (p<0.05) to leave the “start zone”, after the removal of a partition separating the fish from the long arm of the maze (Fig. 9A). Once leaving the “start zone”, fish of the three age groups spent similar amounts of time in the “moving zone” (the initial 2/3 of the long arm of the maze). However, at Baseline, the older fish spent significantly (p<0.05) more time in the “choice zone” (the last 1/3 of the long arm), just before entering the right or left short arm of the maze. This effect was observed in both white/white and red/white arms condition (Fig. 9B). The latency to “choice” was generally reduced following the Conditioning phase and increased following the Extinction phase. However, large inter-individual variability in responses was associated with the lack of statistical significance for group comparisons.

Middle-aged ACHE mutants did not show a significant difference in latency to leave the starting zone, or in latency to “choice” under the white/white condition, compared to young fish. While these older mutant fish demonstrated some increase in latency to “choice” in red/white condition, similar to older wild-type fish, the difference did not reach statistical significance, compared to young ACHE mutants (Fig. 9). The irradiated fish had a significantly increased latency to Start and a strong tendency towards increase in latency to “Choice” (Fig. 9).

#### Alternation patterns in the T-maze

**Figure 10:**
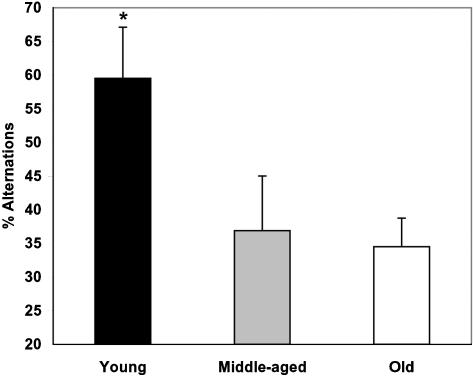
Increased alternation in the consecutive choice of the short arms of the maze during Baseline trials in young zebrafish, compared to middle-aged and old groups. Y-axis: % alternation. * p<0.05, relative to each of the older groups.

Data analysis of fish behavior in the white/white T-maze at Baseline allowed us to assess an alternation pattern for the choice of the short arms. This interesting parameter has been successfully used in rodent research [19] and may represent a degree of stereotypic behavior. Alternation in the choice of the short arms of the maze was found to be significantly higher in young wild-type fish, compared to both middle-aged and old fish (p<0.05 Fig. 10). No significant difference in this parameter was documented between the ACHE mutants, with both age groups being similar to young wild-type zebrafish. As a group, the irradiated fish showed no consistent pattern of arm choice, with some fish demonstrating a highly stereotypic choice of one arm over 14 consecutive trials, while others having showed a high percentage of alternations (data not shown).

### Conditioned place avoidance (CPA) in negative reinforcement paradigm

During the Conditioning phase of the CPA, fish received mild electric shock each time they entered a red-colored half of the tank. Throughout the Extinction period, no electric shock was delivered, although half of the tank remained red.

#### CPA in wild-type zebrafish

**Figure 11:**
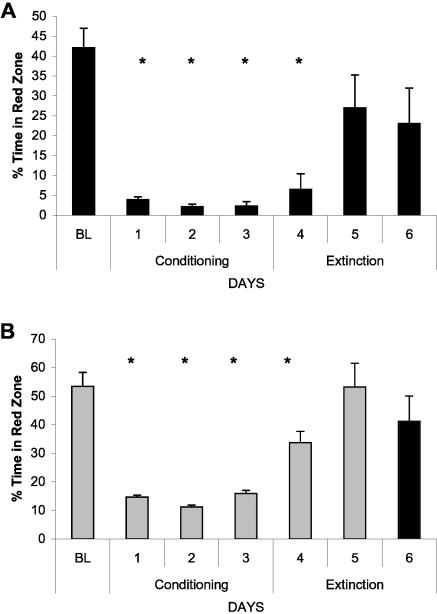
Conditioned place avoidance (CPA) in wild-type young (A) and middle-aged (B) zebrafish. Y-axis: % time spent in the red zone of the test tank at Baseline (BL), during Conditioning and Extinction phases of the study. Conditioning phase: * p<0.00001 for main group and training day effects; no significant difference between age groups. Extinction phase: between group comparison: p<0.0003, <0.0001 and <0.0001 for day 4, 5 and 6, respectively; within middle-aged group comparison, relative to Baseline, p<0.01, NS and NS for day 4, 5 and 6 respectively; within young group, p<0.0001, NS and <0.02 for day 4, 5 and 6, respectively.

This part of the study included young (1 year old) and middle-aged (2 years old) wild-type zebrafish. Under a negative reinforcement paradigm, young and middle-aged fish showed very fast development of avoidance of red color, relative to Baseline (Fig. 11A). No significant difference between the age groups for the Baseline or Conditioning phases was documented. During the Conditioning phase, highly significant (p<0.00001) main group and training day effects were observed and those were sustained over the entire period of Conditioning.

Significant differences between the age groups became apparent during the Extinction phase (between group comparison: p<0.0003, <0.0001 and <0.0001 for day 4, 5 and 6, respectively). On the first day of extinction, the young fish continued avoiding the red zone, while the middle-aged zebrafish significantly (p<0.01) increased the time spent in the red zone and the number of times entering it, compared to the day before (day 4 on Fig. 11B). This age difference remained during the next two days of the Extinction phase, with older animals showing no significant red color avoidance, compared to their Baseline. The young fish increased the time spent in the red half of the tank during the Extinction period, but still significantly avoided the red color area of the tank (p<0.0001 and <0.02 for day 4 and 6, respectively).

#### Generalization of CPA to different environment

**Figure 12:**
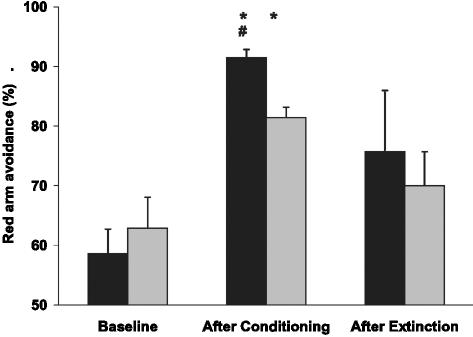
Generalization of red color avoidance in the T-maze condition, following training in negative reinforcement paradigm in young and middle-aged zebrafish. Y-axis: % time young (black) or middle-aged (gray) zebrafish spent outside red color zone of the tank at Baseline, following Conditioning or Extinction phases. *p<0.001, relative to Baseline.

A comparison of zebrafish performance in the T-maze at Baseline and after Conditioning or Extinction phases of CPA, showed that both age groups can generalize a color stimulus, from the environment where it was associated with negative reinforcement to a new experimental paradigm (p<0.01; Fig. 12). Both young and middle-aged zebrafish avoided red color when it was presented in the T-maze following the Conditioning phases of the study, compared to Baseline. This effect was more robust in the young group than in the middle-aged one.

Both the young and middle-aged wild-type zebrafish showed reduced generalization in avoiding the red color during the Extinction phase of the study (Fig. 12). However, increased within-group variability in avoiding red color was found in both groups, reflecting that some fish continued generalizing the CS at the end of the Extinction phase of the experiment, while others stopped avoiding the red color.

## Discussion

The results of the present study show that cognitive abilities in zebrafish undergo age-related changes, with genetic and environmental factors playing an important role in this process. Increases in actelylcholine levels in mutant zebrafish due to altered ACHE activity delay the onset of age-related changes in cognition. In contrast, genotoxic stress of gamma-irradiation accelerates cognitive decline. Thus, zebrafish, a genetically and developmentally well-characterized vertebrate, can be used as a new animal model for studying the genetic background and environmental effects that define the rate of cognitive aging.

The average life-span in outbred zebrafish is reported to be about 42 months, while inbred fish tend to have a 10–15% shorter life-span [20]. Studies on some of the typical signs of aging in 2–3 year old fish (e.g. lipofuscin accumulation, growth rate, proliferation of muscle tissue or telomerase activity) suggest that zebrafish undergo very gradual aging [6], [20]. However, the rate of aging for different physiological systems and functions varies. We have recently found that similarities in growth rate in 1- and 2-year old zebrafish are associated with significant differences in fecundity or increased β-galactosidase activity and decline in melatonin production [16]. While considering zebrafish as a model for human aging, it should also be taken into account that the rate of age-related changes in different systems in mammals and fish may vary. This might result from an adaptation to different environments and, importantly, to a poikilothermic versus a homeothermic type of physiology. The potential discrepancies in the rate of aging in different physiological systems in fish and mammals may become an important source of information, clarifying the roles of specific genes in the aging of individual physiological functions in vertebrates.

The present study compared spontaneous and conditioned behavior in three age groups of wild-type inbred zebrafish, designated as young, middle-aged and old (1, 2 and 3 year of age, respectively). The principal method of assessing fish behavior was by documenting their locomotor activity under different test conditions. Depending on whether the locomotor activity pattern analyzed involved the entire experimental tank, separate areas of the tank or T-maze, this parameter provided information on the overall locomotor activity levels, the degree of place preference or avoidance, latency to initiating T-maze exploration or choice between the arms of the maze. Changes in locomotor activity in such experimental paradigms may potentially reflect inter-group differences in a number of physiological and cognitive functions, including motor ability, learning and memory, motivation and/or some aspects of anxiety or fear, in zebrafish (e.g. neophobia). A combination of the experimental paradigms reported here was aimed at differentiating some of these potential reasons for the behavioral phenotypes.

Compared to young adults (1 year old), the 2-year old and 3-year old inbred (AB strain) wild-type zebrafish have significant changes in locomotor activity. On average, older fish have lower baseline activity levels, reflected in both the total distance traveled and speed. Such changes in locomotor activity manifest in tanks of different sizes, suggesting that this feature is intrinsic rather than environment-dependent. However, among middle-aged and even old fish there were animals with a sustained high level of activity, while some of the young fish had a relatively low activity level. Comparisons of individual locomotor activity levels and cognitive performance within each age group did not show significant correlation in the present study. While the mean group levels support a conclusion of age-related decline in zebrafish locomotor activity with aging, future studies need to assess a potential correlation between individuals' locomotor activity levels at a young age, their changes throughout the life-span, and their decline in cognitive performance with advanced age.

Age-related changes in circadian rhythmicity have become an important area of research, especially since cognitive dysfunctions in human dementias are often associated with circadian rhythm and sleep disorders. A specific nature of the food-entrainable circadian rhythms, and their partial independence from the major circadian clock that is entrained by light, has been established in many studies. However, the mechanisms of this phenomenon remain to be fully elucidated. A few studies on the food entrainment in aged rodents produced conflicting results, some suggesting that this rhythm deteriorates with age [21], [22]. However, others did not confirm this conclusion [23].

Zebrafish have been successfully used for circadian rhythm research, showing that multiple, central and peripheral oscillators can regulate the physiological and behavioral functions in this vertebrate [for review, 11]. However, to our knowledge, the food-entrainable oscillator has not yet been studied in zebrafish. The data obtained in this study support the idea that zebrafish can be an efficient model to explore the mechanisms of food-entrainable circadian rhythms.

According to our results, middle-aged and old wild-type zebrafish are slower in establishing an anticipatory increase in locomotor activity close to the time of regular food administration. While in this study young fish developed significant anticipatory behavior during 7 days of adaptation to a new feeding schedule, the older fish groups showed very gradual and less significant anticipatory increase in activity throughout the study. The anticipatory behavior, however, may reflect several functions, in addition to circadian entrainment, e.g. an appetite level. Further studies, using modified designs and assessing both food- and light-entrainable circadian oscillators, will help to differentiate these options and establish the reasons for the slow development of food anticipation in older zebrafish.

The conditioned place preference or avoidance paradigms are widely used for determining the motivational properties of procedures or drugs. Since the mechanisms of positive and negative reinforcement may differ in some aspects, we chose to test the responses of zebrafish of different ages using both an attractive stimulus (food) and an aversive one (mild electric shock). To make the results of these paradigms easier to compare, we employed the same conditioning stimulus, the red color, that zebrafish can differentiate well [24]. However, based on the preliminary data, we used different sizes of experimental tanks in CPP and CPA procedures, when the CPA tank was much smaller. The smaller tank made it more difficult for the fish to avoid a specific area, since they normally traverse large distances and normally only stay in one place for a prolonged period when they sleep [12]. Using a similar smaller tank for a CPP procedure with food reinforcement makes it difficult to assess a conditioned response for at least two reasons. Zebrafish have a natural trend toward covering large areas while moving. In addition, inevitable fast distribution of food around a small tank due to fish movement reduces the chance of the fish associating a specific tank area with food presentation.

When trained using a positive reinforcement stimulus, it takes older fish longer to develop a conditioned response, with some fish not developing it following 7 days of training. However, conditioning to a negative reinforcement stimulus, shock, developed at a similar rate in young and older fish. This might suggest that older fish can learn quickly but require stronger motivation to acquire a conditioned response. Alternatively, somewhat different mechanisms might be responsible for the learning process associated with negative and positive reinforcement, which could be differentially preserved with aging.

The results of assessing the rate of avoidance of red color in the T-maze, before and after the conditioning phases of the CPA study, show that young and middle-aged zebrafish can generalize a CS presented in a new context and can react adequately. Young fish were also successful in generalizing a CS, following positively reinforced training according to CPP. However, age appears to be an important factor in developing generalization, since the middle-aged and old zebrafish did not develop generalization following CPP procedure. This correlates with the longer period of learning a conditioned response by aged zebrafish in the environment where a CS was positively reinforced. An inverse relationship between age and the degree of generalization, depending on the type of stimulus used, suggests that a stronger US (e.g. an electric shock) increases the chance of CS generalization in older zebrafish.

Extinction of an acquired conditioned response is recognized as an important step in the adaptive modification of behavior in a changing environment [for review, 25]. However, extinction is a complex process, which may include forgetting, new learning or a combination of both. Differentiating these components might not be trivial. For example, an extinction memory may compete with conditioned memory for determining an expression of preference or avoidance behavior. The final behavioral outcome may also depend on the context in which an earlier established CS is introduced, compared to the conditioning and extinction environment. In addition to the defined CS, other environmental cues, not necessarily recognized by the researchers, may facilitate a recall of the CS meaning.

This study demonstrates that extinction of a conditioned response changes with age in zebrafish, and occurs significantly faster in older than in younger fish. However, two distinct extinction protocols were used following the conditioning in CPA and CPP procedures, and they may involve somewhat different mechanisms. The conditioning phase of the positive reinforcement paradigm was followed by presenting the US (food) in the absence of the color or spatial CS, i.e. food was administered into the middle of an all-white tank. Such a design was aimed at encouraging the zebrafish to forget the CS, rather than re-learn its new meaning or absence. In contrast, extinction of a negatively reinforced avoidance response was conducted in the presence of a non-reinforced CS and in the context of high motivation to move into a previously avoided territory in a small tank. Such a classic extinction paradigm is more likely to involve re-learning of a new meaning or lack of a meaning of the CS, though forgetting an earlier association might still be a factor.

In the positive reinforcement paradigm, when the US (food) continues to be presented at the same time of day but in the absence of CS and in a different part of the tank, the younger animals quickly lost their conditioned response to a spatial cue, the side of food administration. In our study, the groups of older fish did not develop this spatial response during CPP Conditioning phase, thus it was not possible to address extinction of the response. A conditioned response to red color in the positive reinforcement paradigm was reduced in young zebrafish, after this color was not present for several days during the food administration. Older fish showed no color preference following the extinction period. Extinction of the negatively reinforced avoidance response, conducted in the presence of a non-reinforced CS and with high motivation to re-establish baseline behavior, resulted in a extinction process that was fast in older fish and slower in young fish.

The results obtained allow only cautious speculations regarding the role of forgetting and re-learning in the extinction processes in zebrafish. At this point, we are inclined to explain a faster reversal of conditioned responses in older fish by their short-lived new memory trace, resulting in quick reversal to baseline behavior in both CPP and CPA paradigms. This could also account for a longer period of learning the CS-US association or for not learning it at all in CPP. Based on these initial data, an extended battery of tests will be used to further characterize the extinction process in zebrafish and its link to learning and re-learning of new skills, in order to determine the forgetting and new learning abilities at different ages.

Importantly, these conclusions regarding the age-related changes in zebrafish cognitive performance should take into account that among middle-aged and older fish groups were individuals that were capable of performing at the level of young zebrafish. While this phenomenon is observed in other species, including humans, zebrafish might provide an important source of information on the role of the environment and genetic phenotype in the aging process. A unique advantage of using zebrafish as models would be the large clutches of eggs (about 200) that can be obtained from each female fish at a time. This provides the possibility for large-scale genetic screens or environmental manipulations in externally fertilized and developed embryos. High throughput behavioral assays can be conducted in several-day old larvae of the same clutch and then in the same fish at different ages. These siblings, wild-type or mutants, can provide abundant material for comparing the role of genetic and environmental factors in the process of cognitive aging.

As an initial step toward this line of research, we have characterized cognitive performance in CPP procedure, followed by a T-maze test, in an ACHE mutant, *ache*sb55/+, and in fish subjected to the genotoxic stress of gamma irradiation at a young age. The results show that both the genetic and the environmental factors can affect cognitive performance in zebrafish. They may either delay or advance the onset of a cognitive aging behavioral phenotype, as assessed in middle-aged or even young fish.

In contrast to wild-type fish, a comparison of 1- and 2-year old ACHE mutants suggests a delay in the onset of cognitive aging. The heterozygous ACHE mutants have been shown to have increased acetylcholine levels in larval and adult stage, due to a point mutation in the acetylcholinesterase gene [15], [26]. These fish have recently been found to have superiority in learning the T-maze paradigm, where the choice of arms is reinforced by food administration, and found to show attenuated behavioral responses to amphetamine [26]. In our study, the middle-aged ACHE mutants showed no significant difference in the learning and extinction curves in the CPP paradigm, as compared to 1-year old mutants or 1-year old wild-type siblings. 2-year old wild-type siblings of ACHE mutants performed similarly to the AB-strain wild-type fish of the same age, and were significantly different from the corresponding 1-year old fish in locomotor activity and in learning curve measures. The mutants of both ages performed similarly on the generalization T-maze test, with one exception. Following the CPP Extinction phase, preference of red color in the T-maze remained significant in young wild-type and middle-aged ACHE mutants, but not in young mutants or middle-aged wild-type fish. A combination of this result with fast learning in young mutants may indicate that they might be able to re-learn faster than the older mutants or young wild-type zebrafish. However, this issue has to be further explored.

Overall, the data collected in mutant fish with high acetylcholine levels may suggest that the cholinergic system plays an important role in the decline of cognitive functions in zebrafish, similar to that shown in humans [27]. A detailed characterization of cholinergic pathways in zebrafish brain [28], found to be similar to those in mammals, makes it even more attractive to study the role of the cholinergic system in cognitive aging in zebrafish.

In contrast to the preserved cognitive performance in aged ACHE mutants described above, animals subjected to genotoxic stress by gamma-irradiation at 6 months of age showed early cognitive deficits 7 months later. Similarly to older non-irradiated wild-type fish, they had reduced locomotor activity, a longer period of learning in the CPP paradigm and a lack of generalization of the conditioned stimulus to a new environmental context of the T-maze. It should be noted that while the generalization was quite similar between the young wild-type groups, the 1-year old wild-type and control-to-irradiated fish, the latter failed to reach a statistically significant retention of color preference in the T-maze following Extinction. Future studies would benefit from a larger number of animals per group in addressing the extinction process in zebrafish.

Previously, we found that irradiation at this age is associated with other signs of premature senescence, including lower reproductive abilities, increased senescence-associated β-galactosidase activity, altered fin regeneration and low melatonin production [16]. However, the same study showed that these signs of early senescence in irradiated fish were accompanied by an unchanged rate of growth. Thus, some but not all normal physiological functions are altered by this strong environmental factor. It would therefore be interesting to assess the potential correlations (or causal dependencies) of the specific changes in cognition, other body functions and regulatory pathways following such genotoxic stress, and assess its dose-dependence.

While analyzing the T-maze performance data, we compared the alternation pattern in arm choice of maze performance, as was first implemented in rodent studies [19]. The Baseline alternation pattern in the T-maze was assessed while both short arms of the maze had the same white color. Such a pattern could not be properly assessed afterwards due to the presence of another experimental variable, i.e. random alternation of the color of the short arms during the post-Conditioning and post-Extinction T-maze test procedures. At Baseline, the older wild-type fish showed increased consecutive choice of the same arm of the maze, compared to young animals. The ACHE mutants of both ages had alternation patterns similar to that of young wild-type fish. In contrast, fish subjected to gamma-irradiation 7 months prior to behavioral testing demonstrated high within-group variability, presenting extreme behaviors of choosing one arm of the maze in all 14 consecutive trials or frequent alternation between the arms.

An alternation pattern is an interesting behavioral parameter, revealing age-dependent changes in zebrafish. However, an interpretation of the results of this test may vary depending on the experimental context and on other types of behaviors demonstrated by the same animal. In the context of our study, we have interpreted high alternation rate as increased exploratory behavior in young and ACHE fish, and low alternation rate in aged animals as the development of stereotypy. However, high alternating pattern in the gamma-irradiated fish may have to be interpreted somewhat differently, since this behavior was associated with increased latency to start, reduced speed of movement and, in some cases, return to the start zone, which was not typical of wild-type or mutant fish. Thus, we have cautiously defined such behavior in irradiated fish as “random”, rather than exploratory, and plan to further investigate the phenomenon in a larger number of irradiated zebrafish.

Based on our results, food entrainment declines with aging in wild-type zebrafish but is attenuated in the animals with genetically-determined upregulation of the cholinergic system. This finding is especially interesting in view of earlier reports that suggest the central cholinergic system is involved in the temporal regulation of feeding behavior, based on pharmacological and neurochemical observations [29, 30.]. Age-related cholinergic dysfunctions in caudal diencephalic and brainstem areas were proposed to be responsible for attenuated food anticipation in aged rats [31].

Furthermore, recent data on the important role of the dorso-medial nuclei (DMN) of the hypothalamus in food entrainment [32] might further support this idea. The pattern of DMN activity correlates with feeding time. The preprandial rise in locomotor activity can be blocked by cell-specific DMH lesions and the degree of food entrainment correlates with the number of remaining DMH neurons. Previously, the activity of the DMN was implicated in increased gut motility mediated, at least in part, through vagal cholinergic pathways [33]. Thus, the lack of age-dependent deterioration in food entrainment in zebrafish mutants with high acetylcholine levels might involve upregulation in the cholinergic input to the central structures and/or effector organs, promoting periodic food-related behaviors.

In summary, the neurobiology of cognitive aging suggests that several factors contribute to the level of cognitive ability in aged individuals, including a significant genetic component and a critical role of environmental and social factors. Animal models in genetically well-characterized species with complex individual and social behaviors can provide critical information on the genetic background of normal and pathological aging of cognitive functions.

The results of this study suggest that aging in zebrafish is associated with cognitive changes, reflected in the speed of learning and extinction, generalization of conditioned stimuli and exploratory versus stereotypic behaviors. The age-related changes in zebrafish cognition manifest as early as 2 years of age and then further deteriorate. Use of mutant and environmentally-affected zebrafish showed potential advantages of studying the genetic and environmental factors that modulate the rate of aging in this animal model. Comparing young and middle-aged fish may allow the assessment of both delayed and accelerated rate of senescence. Thus, using the zebrafish model to study aging may not require waiting until close to the end of a relatively long life span in zebrafish. However, when needed, this fish can help to elucidate critical factors that produce delayed negative or positive effects on cognition. A relatively long life span in zebrafish could also help exploring therapeutic strategies that are applicable to old or very old individuals.

The vast knowledge of zebrafish genetics and molecular biology, multiple well-characterized mutant and transgenic phenotypes, complex and diverse behavioral patterns in a highly social schooling fish, unusually high fecundity among the vertebrate models and the ease of administering experimental drugs, makes this small vertebrate an excellent model for studying cognitive aging throughout the life span.

## Materials and Methods

### Subjects

Male zebrafish (*Danio rerio*) of different ages, wild type (AB strain), *ache*sb55/+ mutants (ACHE) and their wild-type siblings (gift of Dr. Uwe Strähle), were used in these experiments. Prior to the experimental period, fish were housed in multi-tank flow-through systems under standard environmental conditions, including 14∶10 light∶dark cycle and 27°C water temperature, continuous aeration, filtration, ultraviolet sterilizers to disinfect the water and daily water changes (10–15% of volume). Water quality was controlled daily. Fish were maintained in groups, 5–7 fish per 2.8 L tank, and fed newly hatched brine shrimp and TetraMin flake fish food twice a day (at 10AM and 4PM).

At the start of the Adaptation periods, fish were transferred to individual tanks with a mesh-covered top. Individual tanks were placed in 40 L holding tanks with water filtration system and daily 15% water exchange. Prior to feeding time or experimental procedures, individual tanks were moved out of the holding tank, while retaining the water. Food leftovers were removed an hour after feeding, the water was partially changed and tanks were returned into the holding tank. The same or similar individual tanks were used for conducting the experimental procedures, therefore maintaining a constant environment throughout the experimental period and reducing the stress associated with the between-tank transfer.

Wild-type fish groups (7–12 fish each) included young (1.2 years of age), middle-aged (2.1 years of age) and old (3.4 years of age) fish. Within each group, fish were of the same age, i.e. raised from the eggs spawn by several female zebrafish on the same day. Some fish were irradiated (20 Gy) at 6 months of age. These fish and control fish (0 Gy) were 1.1 years of age at the time of the experimental testing described. Groups of ACHE mutants and their wild-type siblings (10 fish per group) included young (1.03 year of age) and middle-aged (2.2 years of age) fish. Zebrafish ACHE mutants [15] harbor a point mutation in the acetylcholinesterase (AChE)-encoding gene, resulting in significant increase in acetylcholine levels in ACHE heterozygotes [15], [23]. The heterozygous fish and wild-type siblings were separated based on the characteristic pattern of developmental abnormalities of the homozygous progeny of these fish, as previously described [15], [23].

### Locomotor activity recordings

Zebrafish locomotor activity in the experimental paradigms described below was documented using VideoTrack image analysis software (View Point, Lyon, France).

In order to avoid potential entrainment of the fish to the visual cues in the distant environment, the recording apparatuses were shielded from the rest of the room with the all-white enclosure. Continuous tracking of each fish documents distance traversed and time spent in fast or slow motion in multiple areas of individual tanks or test apparatus. “Inactivity” was defined as less than 0.1 cm per min, to eliminate a possibility of camera noise or small movements of the fins interfering with the activity analysis. The speed of the fast movement was defined as 14 cm/min, or above. The range between 0.1 and 14 cm/min was considered to be a slow movement. The program conducts this distinction automatically, based on the pre-set thresholds, and the raw data obtained at the end of the experiment presents the results for each type of activity.

### Conditioned place preference (CPP) with positive reinforcement

Fish were transferred to individual tanks (180 × 90 × 75 mm), as described above. Throughout the Adaptation period (7 days), the standard amount of food, pre-soaked decapsulated brine shrimp eggs, was provided once-a-day, at 12:00 h. New timing and feeding fish once a day enabled the study of the development of anticipatory reaction to food administration under increased motivation.

During the Baseline period (4 days), the activity level was documented over the entire tank area and each half of the tank, starting two hours before food administration. The number of times fish entered the right or left half of the tank was also documented. Throughout the Adaptation and Baseline period, food was delivered manually to the middle of each tank and all the walls of the tank were surrounded with a white non-transparent filter.

In addition, on days 3 and 4 of the Baseline period, two hours following daily feeding, the walls of one half of the tank were covered by a red filter (red/white tank). The filter stayed for 15 min while fish activity was recorded. The other part of the tank remained with white walls. No food was administered at that time. This served to evaluate a potential preference or avoidance of red color by individual fish at Baseline.

The Test period consisted of Conditioning and Extinction phases. Starting day 1 of the Test period, the red filter was placed over the walls of half of the tank 5 min prior to food administration. The food was then placed on the red side of the tank (slow delivery through pipette) and the filter remained for an additional 10 min of food consumption. Thus, the red color served as a conditioned stimulus (CS) and food as an unconditioned stimulus (US) (Fig. 1A). This procedure was repeated daily for 7 days of the Conditioning phase (days 1–7). The red filter was always placed on the same side of the tank (right or left for individual animals). Thus, the conditioned stimulus also served as a spatial cue for one side of the tank. The latter was important for evaluating anticipatory side preference prior to CS and food administration. On days 8–11, 4 days of Extinction phase, the fish were fed at the same time (12:00 h), while all the tank walls remained white and food was administered to the middle of the tank. On day 12, red color was introduced on the same side of the tank as during Conditioning for 5 min prior to food administration. This allowed to assess place preference following the Extinction phase.

During all the experimental phases, the percentage of time spent in each side of the tank and distance traversed was analyzed within a) 120 min prior to red color presentation time, in 30-min intervals; b) during 5 min of color presentation prior to food (or corresponding period when red color was not used) and c) 60 min after food was administered (Fig. 1A).

### Conditioned place avoidance (CPA) with negative reinforcement

Throughout the experiment, starting with the Adaptation period, fish were maintained in individual housing tanks (120 × 50 × 50 mm), similar to described above. Following Adaptation to individual tanks, fish were introduced to the test tank, which was identical to the housing tank, except for the presence of four stainless steel flat electrodes, two on each long wall of the tank. Similar to the CPP paradigm, the walls of the test tank were either kept white (white/white condition) or half of the wall perimeter was covered by a red filter (red/white condition). During Baseline data collection, fish activity was recorded in white/white and in red/white condition, with no US used. Throughout the Test period, a 5-min recording was conducted in white/white condition (no US), followed by a 5-min test session in red/white environment (Fig. 1B). On each day of the Test period, the red color was introduced randomly on either half of the tank (“red zone”). During Conditioning, a mild electric shock (5 mV, 0.1 sec ; pulse generator MASTER-8-CP, A.M.P.I, Israel) was delivered each time the fish entered the red zone or every 5 sec if it stayed there, i.e., serving as an US. Thus, the red color on the walls of one half of the tank served as a CS. During the Extinction phase, no electric shock was delivered in the red/white experimental tank containing inactive electrode plates. The time fish spent in each half of the tank and the number of times they entered each side during a test session was recorded.

### Generalization to a Conditioned Stimulus

Recognition of an earlier learned CS in a different environment, where such CS has not been positively or negatively reinforced, is an important cognitive ability, which can increase individual survival. In order to test whether fish can develop generalization to visual CS, they were tested in a Plexiglas T-maze (Fig. 1C). The areas of the T-maze recorded included the starting zone, first 2/3 of the long arm (“movement zone”), last 1/3 of the long arm (“choice zone”), and the right and left short arms. After the fish reached one of the deep end chambers, they would be allowed to remain there for 30 sec. Then, the entire chamber would be lifted and the fish transported back to the starting zone, while continuously remaining in water. Such technique helped to reduce the stress of transfer.

The T-maze had either all-white arms or the front wall of one of the short arms was red (filter insert). Prior to the CPA or CPP tests, i.e., at Baseline, fish were evaluated in white/white and red/white T-maze to control for the behavioral asymmetry, alternations between the arms and red color preference/avoidance. They were then tested in red/white T-maze at the end of the Conditioning and Extinction phases of the CPA or CPP test.

Each T-test procedure included 14 consecutive trials. During the white/red trials, the red filter was introduced randomly either on the left or right arm of the maze at each consecutive trial. The red/white and left/right choice, alternation pattern (i.e., number of entries into an opposite arm of the maze during consecutive trials) and locomotor activity level at several zones of the maze was documented.

### Statistical analysis

The data were analyzed using the StatView (SAS Institute Inc., Cary, NC, USA) or SPSS® (SPSS, Chicago, IL) statistical packages. The activity measurements within the CPP and CPA test sessions (including Baseline, Conditioning and Extinction phases of the experiment) were analyzed using repeated measures ANOVA with two factors: age and zone of activity (e.g., white vs. red zone). T-maze values were analyzed by a one-way ANOVA model with a between age group factor. Bonferroni correction for multiple comparisons and Newman Keuls test for post-hoc comparisons were used when appropriate. The significance level was *p*<0.05. The data presented in the graphs reflect means and standard error of the mean for each group.

The experimental protocol was approved by the Boston University Institutional Animal Care and Use Committee.

## References

[1] Sarter M, Bruno JP (2004). Developmental origins of the age-related decline in cortical cholinergic function and associated cognitive abilities.. Neurobiol Aging.

[2] Craik FI, Bialystok E (2006). Cognition through the lifespan: mechanisms of change.. Trends Cogn Sci.

[3] Schaffitzel E, Hertweck M (2006). Recent aging research in Caenorhabditis elegans.. Exp Gerontol.

[4] Peters A (2002). The effects of normal aging on myelin and nerve fibers: a review.. J Neurocytol.

[5] Gerhard GS (2003). Comparative aspects of zebrafish (Danio rerio) as a model for aging research.. Exp Gerontol.

[6] Kishi S (2004). Functional aging and gradual senescence in zebrafish.. Ann N Y Acad Sci.

[7] Keller ET, Murtha JM (2004). The use of mature zebrafish (Danio rerio) as a model for human aging and disease.. Comp Biochem Physiol C Toxicol Pharmacol.

[8] Delaney M, Follet C, Ryan N, Hanney N, Lusk-Yablick J (2002). Social interaction and distribution of female zebrafish (Danio rerio) in a large aquarium.. Biol Bull.

[9] Engeszer RE, Ryan MJ, Parichy DM (2004). Learned social preference in zebrafish.. Curr Biol.

[10] Larson ET, O'Malley DM, Melloni RH (2006). Aggression and vasotocin are associated with dominant-subordinate relationships in zebrafish.. Behav Brain Res.

[11] Cahill GM (2002). Clock mechanisms in zebrafish.. Cell Tissue Res.

[12] Zhdanova IV, Wang SY, Leclair OU, Danilova NP (2001). Melatonin promotes sleep-like state in zebrafish.. Brain Res.

[13] Bartus RT, Dean RL, Beer B, Lippa AS (1982). The cholinergic hypothesis of geriatric memory dysfunction.. Science.

[14] Raza A, Milbrandt JC, Arneric SP, Caspary DM (1994). Age-related changes in brainstem auditory neurotransmitters: Measures of GABA and acetylcholine function.. Hear Res.

[15] Behra M, Cousin X, Bertrand C, Vonesch JL, Biellmann D (2002). Acetylcholinesterase is required for neuronal and muscular development in the zebrafish embryo.. Nat Neurosci.

[16] Tsai SB, Tucci V, Uchiyama J, Fabian, Lin M, Zhdanova IV, Kishi S (2006). Differential effects of genotoxic stress on both concurrent body growth and gradual senescence in the adult zebrafish.. Aging Cell.

[17] Bolles RC, Stokes LW (1965). Rat's anticipation of diurnal and a-diurnal feeding.. J Comp Physiol Psychol.

[18] Schibler U, Ripperger J, Brown SA (2003). Peripheral circadian oscillators in mammals: time and food.. J Biol Rhythms.

[19] Lalonde R (2002). The neurobiological basis of spontaneous alternation.. Neurosci Biobehav Rev.

[20] Gerhard GS, Cheng KC (2002). A call to fins! Zebrafish as a gerontological model.. Aging Cell.

[21] Tanaka Y, Kurasawa M, Nakamura K (2000). Recovery of diminished mealtime-associated anticipatory behavior by aniracetam in aged rats.. Pharmacol Biochem Behav.

[22] Mistlberger RE, Houpt TA, Moore-Ede MC (1990). Effects of aging on food-entrained circadian rhythms in the rat.. Neurobiol Aging.

[23] Walcott EC, Tate BA (1996). Entrainment of aged, dysrhythmic rats to a restricted feeding schedule.. Physiol Behav.

[24] Robinson J, Schmitt EA, Harosi FI, Reece RJ, Dowling JE (1993). Zebrafish ultraviolet visual pigment: absorption spectrum, sequence, and localization.. Proc Natl Acad Sci USA.

[25] Myers KM, Davis M (2002). Behavioral and neural analysis of extinction.. Neuron.

[26] Ninkovic J, Folchert A, Makhankov YV, Neuhauss SC, Sillaber I (2006). Genetic identification of AChE as a positive modulator of addiction to the psychostimulant D-amphetamine in zebrafish.. J Neurobiol.

[27] Bartus RT (2000). On neurodegenerative diseases, models, and treatment strategies: lessons learned and lessons forgotten a generation following the cholinergic hypothesis.. Exp Neurol.

[28] Mueller T, Vernier P, Wullimann MF (2004). The adult central nervous cholinergic system of a neurogenetic model animal, the zebrafish Danio rerio.. Brain Res.

[29] Ono M, Minamoto Y, Shibata S, Watanabe S (1995). Attenuating effect of arecoline and physostigmine on an impairment of mealtime-associated activity rhythm in old rats.. Physiol Behav.

[30] Inglis FM, Day JC, Fibiger HC (1994). Enhanced acetylcholine release in hippocampus and cortex during the anticipation and consumption of a palatable meal.. Neuroscience.

[31] Mistlberger RE (1994). Circadian food-anticipatory activity: Formal models and physiological mechanisms.. Neurosci Biobehav Rev.

[32] Gooley JJ, Schomer A, Saper CB (2006). The dorsomedial hypothalamic nucleus is critical for the expression of food-entrainable circadian rhythms.. Nat Neurosci.

[33] Greenwood B, DiMicco JA (1995). Activation of the hypothalamic dorsomedial nucleus stimulates intestinal motility in rats.. Am J Physiol 268 Gastrointest Liver Physio.

